# Machine learning derived model for the prediction of bleeding in dual antiplatelet therapy patients

**DOI:** 10.3389/fcvm.2024.1402672

**Published:** 2024-10-02

**Authors:** Yang Qian, Lei Wanlin, Wang Maofeng

**Affiliations:** ^1^ Department of Pharmacy, Affiliated Dongyang Hospital, Wenzhou Medical University, Dongyang, Zhejiang, China; ^2^ Department of Biomedical Sciences Laboratory, Affiliated Dongyang Hospital, Wenzhou Medical University, Dongyang, Zhejiang, China

**Keywords:** dual antiplatelet therapy, bleeding, machine learning, predictive model, nomogram, risk factor

## Abstract

**Objective:**

This study aimed to develop a predictive model for assessing bleeding risk in dual antiplatelet therapy (DAPT) patients.

**Methods:**

A total of 18,408 DAPT patients were included. Data on patients’ demographics, clinical features, underlying diseases, past history, and laboratory examinations were collected from Affiliated Dongyang Hospital of Wenzhou Medical University. The patients were randomly divided into two groups in a proportion of 7:3, with the most used for model development and the remaining for internal validation. LASSO regression, multivariate logistic regression, and six machine learning models, including random forest (RF), k-nearest neighbor imputing (KNN), decision tree (DT), extreme gradient boosting (XGBoost), light gradient boosting machine (LGBM), and Support Vector Machine (SVM), were used to develop prediction models. Model prediction performance was evaluated using area under the curve (AUC), calibration curves, decision curve analysis (DCA), clinical impact curve (CIC), and net reduction curve (NRC).

**Results:**

The XGBoost model demonstrated the highest AUC. The model features were comprised of seven clinical variables, including: HGB, PLT, previous bleeding, cerebral infarction, sex, Surgical history, and hypertension. A nomogram was developed based on seven variables. The AUC of the model was 0.861 (95% CI 0.847–0.875) in the development cohort and 0.877 (95% CI 0.856–0.898) in the validation cohort, indicating that the model had good differential performance. The results of calibration curve analysis showed that the calibration curve of this nomogram model was close to the ideal curve. The clinical decision curve also showed good clinical net benefit of the nomogram model.

**Conclusions:**

This study successfully developed a predictive model for estimating bleeding risk in DAPT patients. It has the potential to optimize treatment planning, improve patient outcomes, and enhance resource utilization.

## 1 Introduction

Dual antiplatelet therapy (DAPT) is a commonly used treatment for patients at risk of cardiovascular events (1). However, one significant challenge associated with DAPT is an increased risk of bleeding complications in some patients (2). One major hurdle is striking the delicate balance between reducing the risk of cardiovascular events and minimizing bleeding risks (3). Overall, although DAPT has proven effective in preventing cardiovascular events, addressing the associated bleeding risk and optimizing patient management remain ongoing challenges in clinical practice.

Several bleeding risk scores have been created to aid in selecting the appropriate treatment regimen and duration. These studies have explored different aspects of bleeding risk assessment, offering valuable insights for clinical practice (4). The ACC/AHA guidelines on DAPT duration were issued prior to the development and validation of the PRECISE-DAPT risk score, a 5-item scoring system, was developed to predict bleeding risk in patients undergoing DAPT (2). It has received a Class IIB recommendation for identifying high-risk patients prone to bleeding. The DAPT score effectively stratifies bleeding and ischemic risk in various study populations, providing the advantage of benefit-risk difference stratification consistently (5). The ESC and EACTS guidelines emphasized a personalized approach to balancing bleeding and ischemic risks, moving away from a generalized strategy in the context of DAPT (6). While American and European guidelines primarily recommend DAPT and PRECISE-DAPT scores, their limitations arise from variations in patient cohorts (7, 8). Recently, new clinical models have been developed and validated in diverse clinical scenarios to improve the prediction of hemorrhagic events. Commonly used scoring systems including PARIS (9), CRUSADE (10), ARC-HBR (11), ACUITY-HORIZONS (12), BleeMACS (13), TIMI risk score (14), HAS-Bled score (15), GRACE score (10), and CHA2DS2-VASC score (16) are extensively employed in clinical practice. These scores assess various clinical characteristics such as coronary anatomy, surgical procedures, genotyping, lifestyle factors, and adherence to treatment (17). Each score has its advantages and limitations based on the characteristics of the patient cohorts used for development and validation, making them applicable to specific patients, clinical contexts, and timeframes (17). such as the TIMI risk score, which is primarily used for assessing risk in patients with non-ST elevation myocardial infarction, and the PARIS score, which is specifically designed for evaluating bleeding risk after coronary interventions. While these tools have broad applicability in their respective domains, they may not fully account for certain unique clinical variables and patient characteristics present in our specific population.

Therefore, our research aims to develop a novel prediction model that addresses this gap. With a growing trend towards individualized bleeding risk stratification for patients receiving DAPT, alternative risk scores validated in large patient cohorts could be applied in specific clinical scenarios that closely resemble those studied.

Further studies on bleeding risk scores are necessary to establish the correlation between bleeding events and the use of these clinical tools. The aim of this study was to create novel bleeding risk scores and evaluate their predictive performance.

## 2 Methods

### 2.1 Study population

Participants in the study were recruited from Affiliated Dongyang Hospital of Wenzhou Medical University. Inclusion criteria for participants were: (1) age over 18 years; (2) The documented utilization of dual antiplatelet therapy (aspirin and clopidogrel) was extracted from hospital electronic medical records (EMRs) spanning the period from January 2008 to December 2017. Exclusion Criteria: (1) Individuals younger than 18 years; (2) Pregnant or lactating women; (3) Patients with incomplete medical histories or examination test results; (4) Patients with missing data on dual antiplatelet therapy (DAPT) or lacking relevant bleeding records; (5) Individuals who died during hospitalization. The study protocol received ethics approval from the Ethics Committee of Affiliated Dongyang Hospital of Wenzhou Medical University (approval #2023-YX-408). Informed consent was waived for this study. Prior to conducting the analysis, all patient medical information was anonymized and de-identified.

### 2.2 Outcome definition

The primary endpoint of this study was defined as the occurrence of any documented bleeding incidents, including gastrointestinal bleeding, intracranial hemorrhage, and other bleeding events such as urinary bleeding, oral bleeding, and ophthalmic hemorrhage (18), within five years following the initiation of dual antiplatelet therapy (DAPT), as recorded in the hospital EMRs at the time of patient discharge. For the purposes of this analysis, the presence of any bleeding event was classified as a positive outcome, while the absence of such events was categorized as a negative outcome.

### 2.3 Risk factors

We obtained the following information from the EMRs of the subjects in our hospital: sex, age, height, weight, BMI, and past medical history, including smoking, drinking, diabetes, hypertension, surgical history, previous bleeding episodes, presence of tumors, acute myocardial infarction, Percutaneous Coronary Interventions (PCI), gastric ulcers, use of gastric protective drugs, cerebral infarction, portal hypertension, anticoagulant usage, and various clinical test indicators such as cardiac ejection fraction (EF), white blood cell count (WBC), platelet count (PLT), peripheral hemoglobin (HGB), and glomerular filtration rate (GFR). For the research parameter, we considered the lowest clinical test indicators within one month prior to commencing DAPT. Other past medical histories were recorded if they occurred before the initiation of DAPT.

### 2.4 Data pre-processing

The data obtained from the clinical research big data platform underwent effective cleaning processes, including the removal of extreme values and imputation of missing values. Indicators with missing values exceeding 20%, such as height, weight, BMI, EF, and GFR, were excluded from the analysis. For the remaining missing predictor values, multiple imputation techniques were employed. To perform model development and evaluation, the data was split into a development cohort comprising 70% of the data and a validation cohort containing the remaining portion. The classification model was trained using the development cohort, while the validation cohort was utilized to assess the model's performance.

### 2.5 Model building

LASSO regression (19) and six machine learning algorithms were utilized to identify the optimal predictive features. Machine learning algorithms employed included random forest (RF), k-nearest neighbor imputing (KNN), decision tree (DT), extreme gradient boosting (XGBoost), light gradient boosting machine (LGBM), and Support Vector Machine (SVM). Shapley additive explanation (SHAP) values were used to identify feature importance. Logistic regression modeling was performed on the 5 or 10 most significant parameters from the best machine learning model, as well as the parameters selected by LASSO regression. These parameters were categorized into three models. To compare the performance of these three models, metrics such as the area under the receiver operating characteristic (ROC) curve (AUC), Net Reclassification Improvement (NRI), and Integrated Discrimination Improvement (IDI) were evaluated. Based on the assessment of the performance metrics, the best model was selected. Using this model, a nomogram for predicting bleeding was established.

### 2.6 Model evaluation

**Figure 1:**
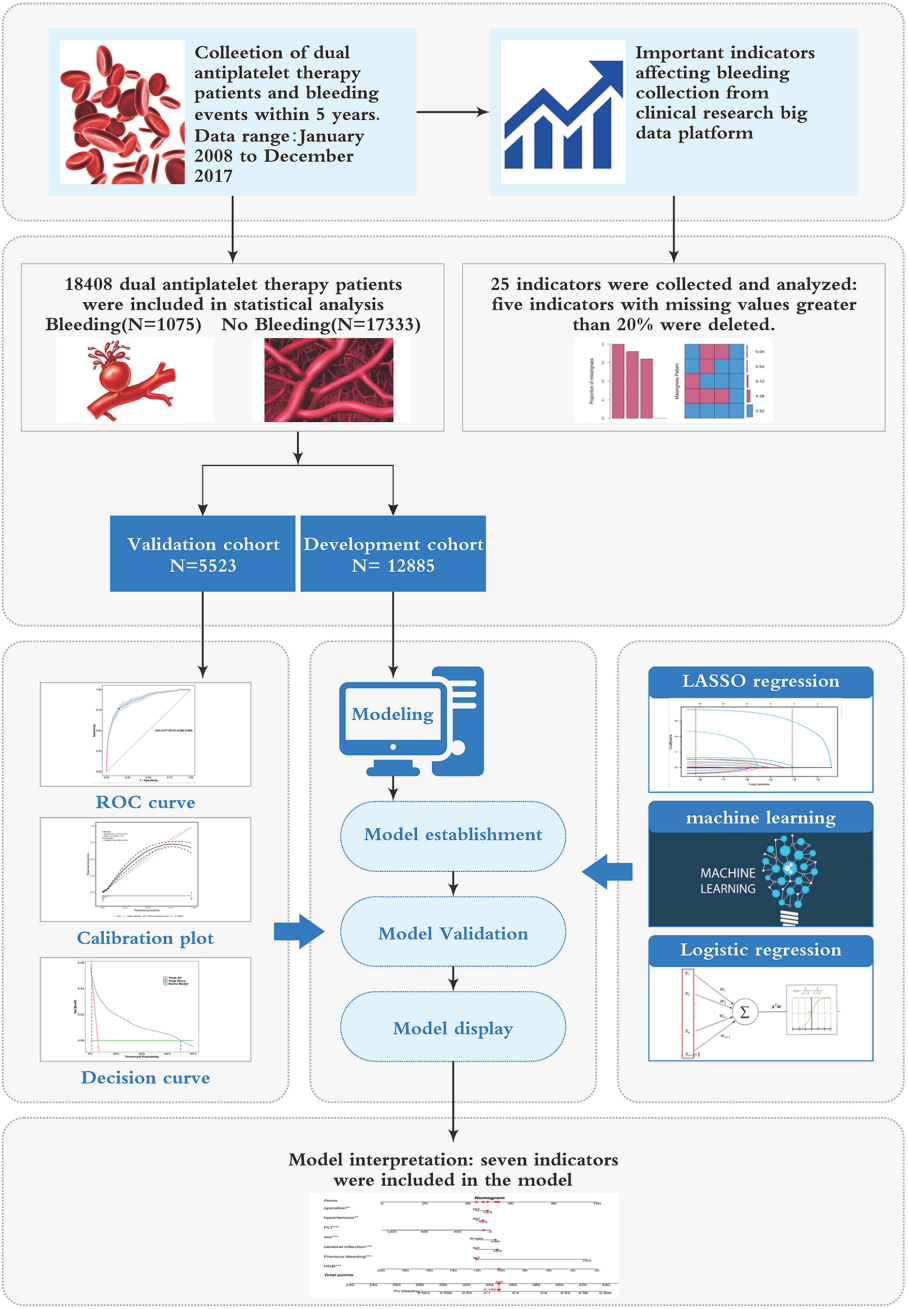
Study process flowchart.

The sensitivity and specificity of the model were assessed using the AUC of the ROC curve, evaluating its discrimination performance. Calibration was evaluated by analyzing calibration curves. The clinical efficacy of the identified risk factors in predicting bleeding risk was verified through decision curve analysis (DCA), clinical impact curve (CIC), and net reduction curve (NRC). These analyses considered the net benefit under varying risk thresholds for patients. Additionally, the model was validated by comparing it to individual indicators in terms of discrimination and clinical utility. Figure 1 illustrates the flowchart outlining the process of model construction and validation.

### 2.7 Statistical methods

Statistical analysis and plotting were conducted using R4.2.1 software for Windows. Categorical variables were presented as frequencies with percentages and compared using χ^2^ test or Fisher's exact test. Continuous variables were expressed as means with standard deviations or medians with interquartile ranges and compared using Student's *t*-test or Mann-Whitney *U* test. Multiple imputation techniques using the “mice” package. Baseline description and differences analysis utilized the “comparegroups” package. LASSO regression employed the “glmnet” package, while multivariable logistic regression used the “glm” package. Discrimination analysis was performed using the “pROC,” “ggROC,” and “fbroc” packages. Calibration assessment utilized the “rms” and “riskregression” packages. DCA and CIC were conducted using the “rmda,” “dca.R,” and “dcurves” packages. The nomogram was created using the “regplot” package, and NRI calculations employed the “nricens” package. IDI analysis was performed using the “PredictABEL” package. Comparisons of multiple models for ROC analysis were conducted using the “ROCR” package, while DCA comparisons were carried out using the “Dcurves” package. Diagnostic evaluation utilized the “reportROC” package. All statistical tests were 2-sided, and a significance level of *P* < 0.05 was considered statistically significant.

## 3 Results

### 3.1 Study population characteristics

A total of 18,408 DAPT patients (clopidogrel and aspirin) were included in this study, among whom 1,075 experienced bleeding (Figure 1). Out of the 25 variables examined, only WBC, HGB, PLT, Height, Weight, BMI, EF, and GFR were continuous variables. We excluded five variables (Height, Weight, BMI, EF, and GFR) with missing information in more than 20% of patients, resulting in 20 variables with missing data in less than 20% of patients (details provided in Supplementary Material S1). There were no significant differences observed in terms of myocardial infarction and PCI between the cohorts with bleeding and without bleeding. Table 1 presents the baseline characteristics of the DAPT patients. A random 7:3 division was performed to allocate patients into the development cohort (*n* = 12,885) and the validation cohort (*n* = 5,523). Table 2 displays the baseline characteristics of patients in both cohorts, revealing no significant differences in each indicator between the two cohorts.

**Table 1:** Baseline characteristics of subjects.

Variables	Total *N* = 18,408	No bleeding *N* = 17,333	Bleeding *N* = 1075	*P*
Sex, *n* (%)				<0.001
Female	7,310 (39.7%)	6,951 (40.1%)	359 (33.4%)	
Male	11,098 (60.3%)	10,382 (59.9%)	716 (66.6%)	
Age (years)	67.0 [57.0;75.0]	66.0 [57.0;75.0]	72.0 [64.0;79.0]	<0.001
Smoke, *n* (%)				<0.001
Yes	12,512 (68.0%)	11,694 (67.5%)	818 (76.1%)	
Drink, *n* (%)				<0.001
Yes	12,512 (68.0%)	11,694 (67.5%)	818 (76.1%)	
DM, *n* (%)				0.003
Yes	2,564 (13.9%)	2,381 (13.7%)	183 (17.0%)	
Hypertension, *n* (%)				<0.001
Yes	8,667 (47.1%)	8,010 (46.2%)	657 (61.1%)	
Surgical history, *n* (%)				<0.001
Yes	839 (4.56%)	736 (4.25%)	103 (9.58%)	
Tumor, *n* (%)				0.002
Yes	448 (2.43%)	406 (2.34%)	42 (3.91%)	
MI, *n* (%)				0.551
Yes	2,028 (11.0%)	1,916 (11.1%)	112 (10.4%)	
PCI, *n* (%)				0.919
Yes	242 (1.31%)	227 (1.31%)	15 (1.40%)	
Previous bleeding, *n* (%)				0.000
Yes	339 (1.84%)	96 (0.55%)	243 (22.6%)	
WBC (10^9^/L)	5.05 [4.13;6.12]	5.09 [4.18;6.15]	4.40 [3.59;5.43]	<0.001
HGB(g/L)	123 [109;135]	124 [110;136	95.0 [74.0;116]	<0.001
PLT (10^9^/L)	166 [131;205]	168 [133;206]	138 [101;176]	<0.001
Gastric protective medicine, *n* (%)				<0.001
Yes	6,341 (34.4%)	5,809 (33.5%)	532 (49.5%)	
Gastric ulcer, *n* (%)				<0.001
Yes	195 (1.06%)	159 (0.92%)	36 (3.35%)	
Cerebral infarction, *n* (%)				<0.001
Yes	7,567 (41.1%)	6,868 (39.6%)	699 (65.0%)	
Portal hypertension, *n* (%)				0.019
Yes	4 (0.02%)	2 (0.01%)	2 (0.19%)	
Anticoagulants, *n* (%)				0.354
Yes	4,561 (24.8%)	4,277 (24.7%)	284 (26.4%)	

DM, diabetes mellitus; MI, myocardial infarction; PCI, percutaneous coronary intervention; WBC, white blood cell count; HGB, hemoglobin; PLT, platelet count.

**Table 2:** The baseline characteristics of the development and validation cohort.

Variables	Total *N* = 18,408	Validation *N* = 5,523	Development *N* = 12,885	*P*
Sex, *n* (%)				0.928
Female	7,310 (39.7%)	2,190 (39.7%)	5,120 (39.7%)	
Male	11,098 (60.3%)	3,333 (60.3%)	7,765 (60.3%)	
Age (years)	67.0 [57.0;75.0]	66.0 [57.0;75.0]	67.0 [58.0;75.0]	0.114
Smoke, *n* (%)				0.082
Yes	12,512 (68.0%)	3,703 (67.0%)	8,809 (68.4%)	
Drink, *n* (%)				0.082
Yes	12,512 (68.0%)	3,703 (67.0%)	8,809 (68.4%)	
DM, *n* (%)				0.650
Yes	2,564 (13.9%)	759 (13.7%)	1,805 (14.0%)	
Hypertension, *n* (%)				0.800
Yes	8,667 (47.1%)	2,592 (46.9%)	6,075 (47.1%)	
Surgical history, *n* (%)				0.952
Yes	839 (4.56%)	253 (4.58%)	586 (4.55%)	
Tumor, *n* (%)				0.924
Yes	448 (2.43%)	133 (2.41%)	315 (2.44%)	
MI, *n* (%)				0.839
Yes	2,028 (11.0%)	604 (10.9%)	1,424 (11.1%)	
PCI, *n* (%)				0.198
Yes	242 (1.31%)	63 (1.14%)	179 (1.39%)	
Previous bleeding, *n* (%)				0.791
Yes	339 (1.84%)	99 (1.79%)	240 (1.86%)	
WBC (10^9^/L)	5.05 [4.13;6.12]	5.06 [4.14;6.13]	5.05 [4.13;6.12]	0.418
HGB(g/L)	123 [109;135]	123 [109;136]	123 [109;135]	0.181
PLT (10^9^/L)	166 [131;205]	166 [132;205]	166 [131;205]	0.950
Gastric protective medicine, *n* (%)				0.542
Yes	6,341 (34.4%)	1,884 (34.1%)	4,457 (34.6%)	
Gastric ulcer, *n* (%)				0.208
Yes	195 (1.06%)	50 (0.91%)	145 (1.13%)	
Cerebral infarction, *n* (%)				0.74
Yes	7,567 (41.1%)	2,260 (40.9%)	5,307 (41.2%)	
Portal hypertension, *n* (%)				0.324
Yes	4 (0.02%)	0 (0.00%)	4 (0.03%)	
Anticoagulants, *n* (%)				0.162
Yes	4,561 (24.8%)	1,420 (25.7%)	3,141 (24.4%)	

DM, diabetes mellitus; MI, myocardial infarction; PCI, percutaneous coronary intervention; WBC, white blood cell count; HGB, hemoglobin; PLT, platelet count.

### 3.2 Selected predictors and construction model

**Figure 2:**
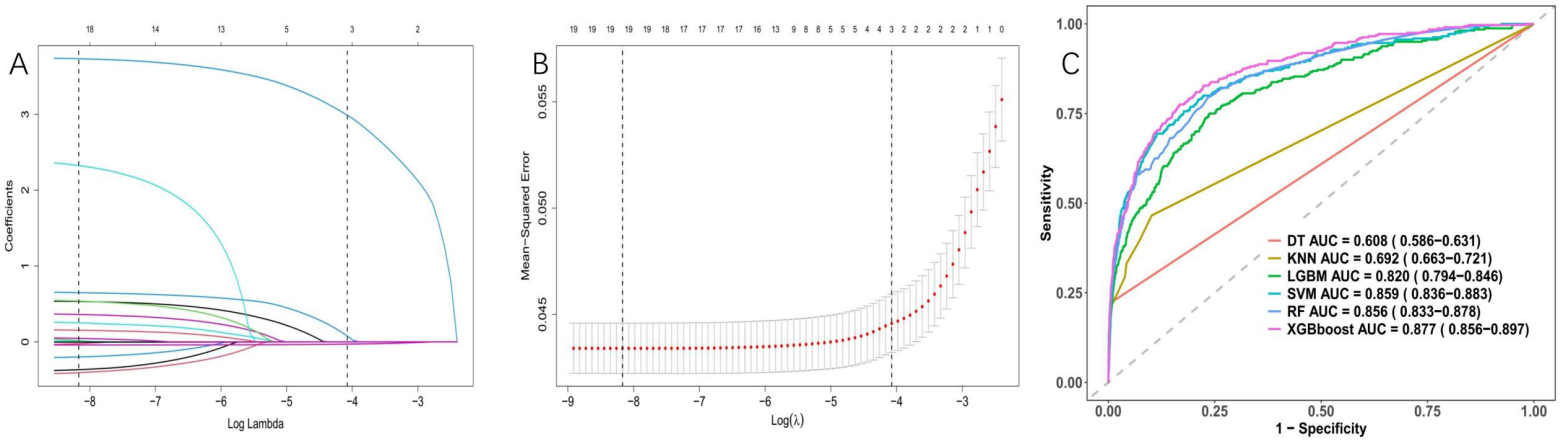
Variable selection was performed using LASSO binary logistic regression and optimal machine learning model selection. **(A)** Coefficient profile plots were generated against the log(lambda) sequence to visualize the variable selection process and identify nonzero coefficient variables based on the optimal lambda value. **(B)** Dotted vertical lines represent optimal values determined using the 1 standard error of the minimum criteria (lambda.1se). **(C)** The right plots display the AUC values of various machine learning models.

**Figure 3:**
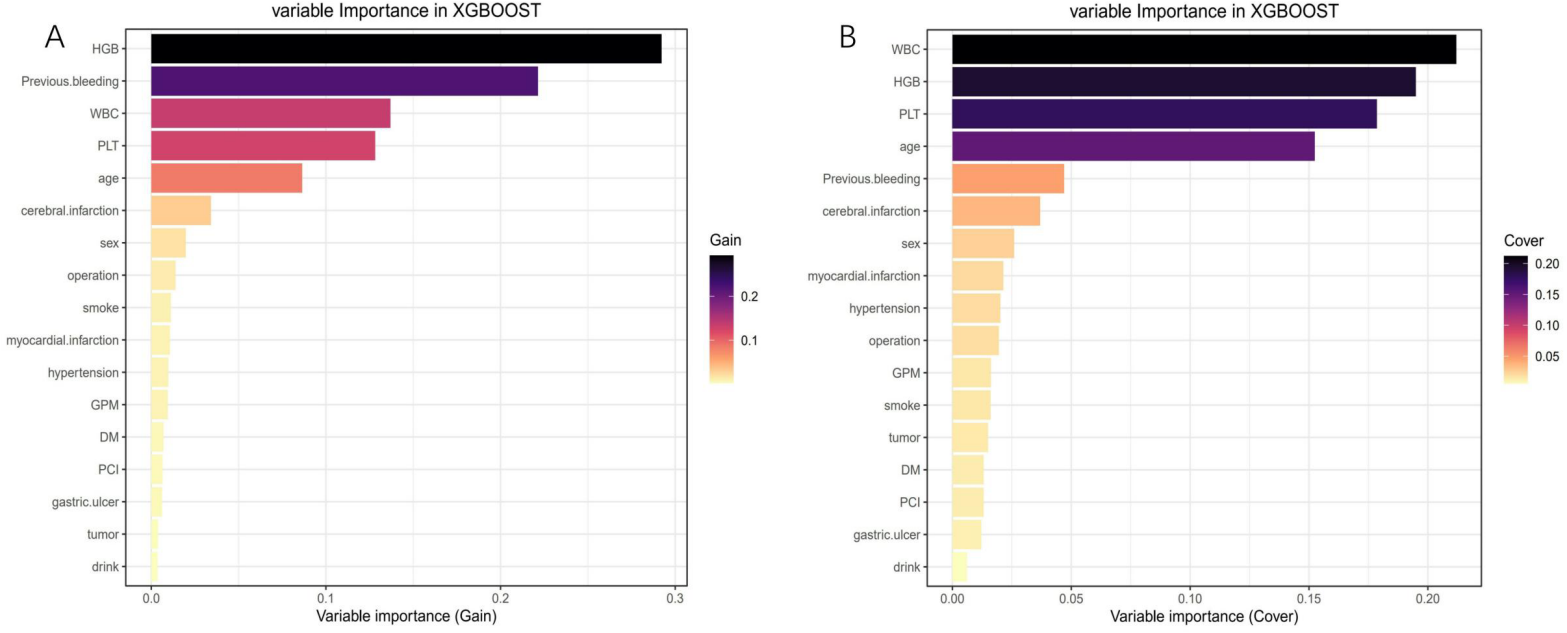
Feature importance analysis using the shapley additive explanation (SHAP). **(A)** Variable importance (Gain); **(B)** Variable importance (Cover).

After conducting LASSO regression with ten-fold cross-validation, three variables (previous bleeding, HGB, and cerebral infarction) were selected for inclusion in the model based on the criteria of “family = binomial” and lambda.1SE. The pathway of variable shrinkage and cross-validation is illustrated in Figures 2A,B, respectively. Additionally, six machine learning models were used to identify the best predictive feature. Among these models, the XGBoost model demonstrated the highest AUC [0.877 (95% CI: 0.856–0.897), Figure 2C], supporting its selection as the optimal model for assessing bleeding risk in DAPT patients. The XGBoost model was further used to explain feature importance using SHAP. Figures 3A,B display important variables based on two methods of variable importance, Gain and Cover, respectively. We selected the five most important indicators, the ten most important indicators in SHAP, and three indicators selected by LASSO regression to perform separate logistic regression modeling. After multivariate logistic regression with the “backward” process, several variables were included in the final models (Table 3). Model 3 included three indicators, while Model 7 included seven indicators.

**Table 3:** Comparison of discrimination between different models.

Model	Include parameters	AUC (95%CI)
Mod Lasso	Previous.bleeding + HGB + cerebral.infarction	0.849 95%CI (0.834–0.864)
Mod 3	HGB + PLT + Previous.bleeding	0.845 95%CI (0.83–0.86)
Mod 7	HGB + PLT + Previous.bleeding + cerebral.infarctio*n* + sex + Surgical history + hypertension	0.861 95%CI (0.847–0.875)

HGB, hemoglobin; PLT, platelet count.

**Figure 4:**
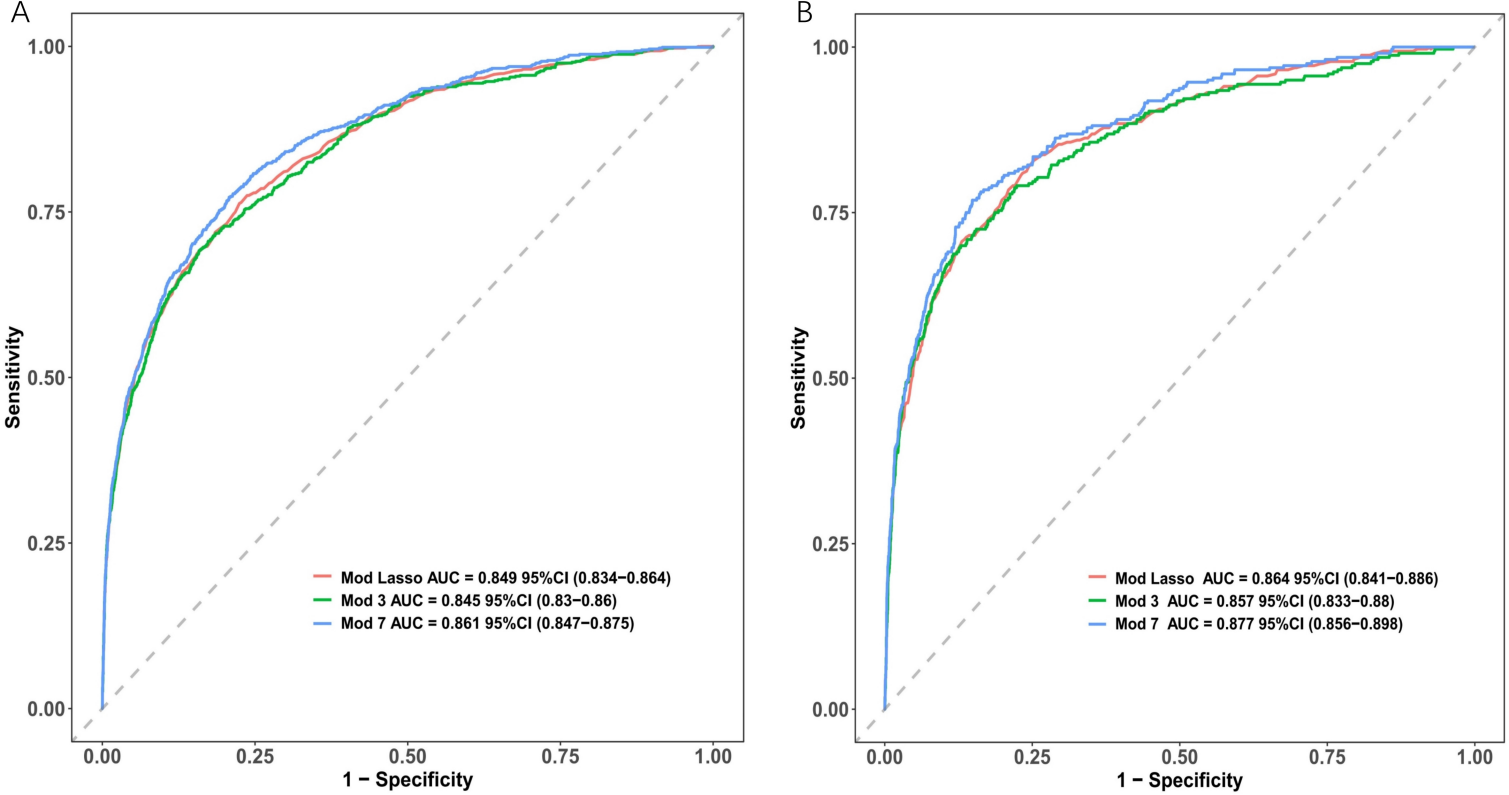
Receiver operating characteristic curves (ROC) of different model distinguishing bleeding from non-bleeding. **(A)** in the development cohort; **(B)** in the validation cohort.

The AUC of the ROC curves for LASSO model, Model 3, and Model 7 in the development and validation cohorts are presented in Figure 4. These results indicate that the discrimination of Model 7 was significantly superior to the other two models according to DeLong's test (*p* < 0.001). Moreover, a comparison of Model 7 and Model 3 performance using NRI and IDI showed that Model 7 exhibited significantly higher values, indicating improved efficacy compared to Model 3 (Table 4). Finally, a nomogram model (Table 5) was constructed using seven variables: HGB, PLT, previous bleeding, cerebral infarction, sex, Surgical history, and hypertension.

**Table 4:** Comparison of NRI and IDI between two models.

	Model 3		*P*
Model 7	NRI (Categorical) [95% CI] [0,0.3) [0.3,1]	0.0358 [0.0185–0.053]	<0.001
	NRI(Continuous) [95% CI]	0.4288 [0.3562–0.5014]	<0.001
	IDI [95% CI]	0.0133 [0.0089–0.0178]	<0.001

**Table 5:** Final model coefficients.

Characteristics	B	SE	OR	CI	*P*
(Intercept)	0.994	0.21261	2.702	1.779–4.096	<0.001
HGB	−0.04	0.00184	0.961	0.957–0.964	<0.001
PLT	−0.003	0.00079	0.997	0.995–0.998	<0.001
Previous bleeding	3.749	0.16785	42.481	30.70–59.32	<0.001
Cerebral infarction	0.696	0.09071	2.006	1.680–2.398	<0.001
Sex	0.54	0.09195	1.717	1.435–2.058	<0.001
Surgical history	0.389	0.15447	1.476	1.082–1.984	0.012
Hypertension	0.218	0.08939	1.244	1.044–1.482	0.015

HGB, hemoglobin; PLT, platelet count.

### 3.3 Model visualization

**Figure 5:**
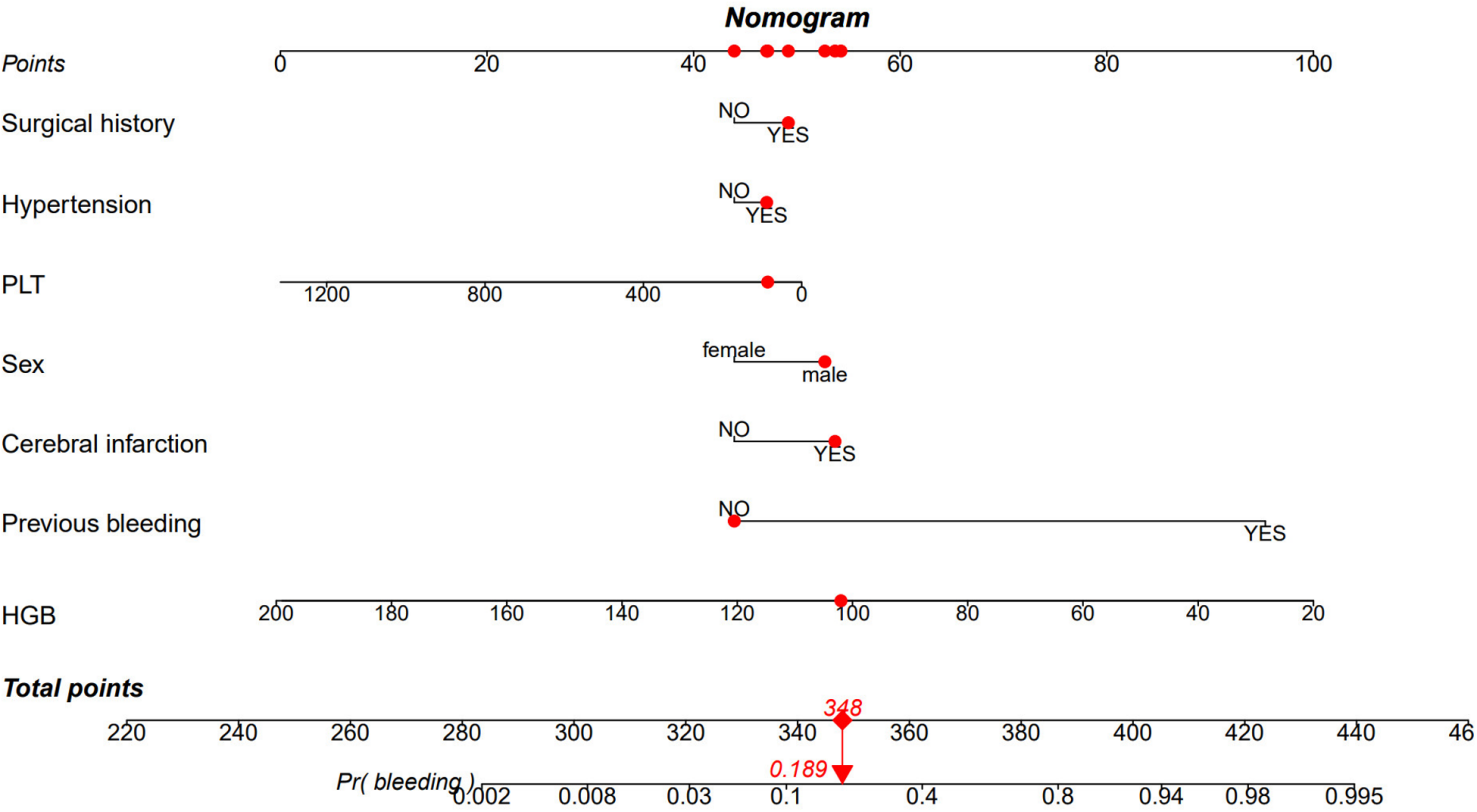
Nomogram based on the combination of seven indicators was developed using logistic regression analysis. If a patient's total score is 348, the corresponding probability of bleeding is 0.189 (highlighted in red). HGB, Hemoglobin; PLT, platelet count.

The nomogram in Figure 5 provides a visual representation of the logistic regression analysis results, enabling the prediction of bleeding risk in DAPT patients. By locating the value of each risk factor on the respective vertical line, points can be obtained. The total points on the nomogram are calculated by summing up the points from each risk factor. To determine the bleeding prediction for a specific DAPT patient, a vertical line is drawn from the total points axis, intersecting with the corresponding probability on the nomogram. For instance, if a male DAPT patient has undergone surgery, has hypertension, developed a cerebral infarction, had previous bleeding, a platelet counts of 86 × 10^9^/L, and an HGB level of 102 g/L, the total score would be 348. Drawing a vertical line from the total score of 348 intersects the probabilit*y* axis at approximately 0.189, indicating an estimated probability of bleeding of 18.9%.

### 3.4 Model validation

**Figure 6:**
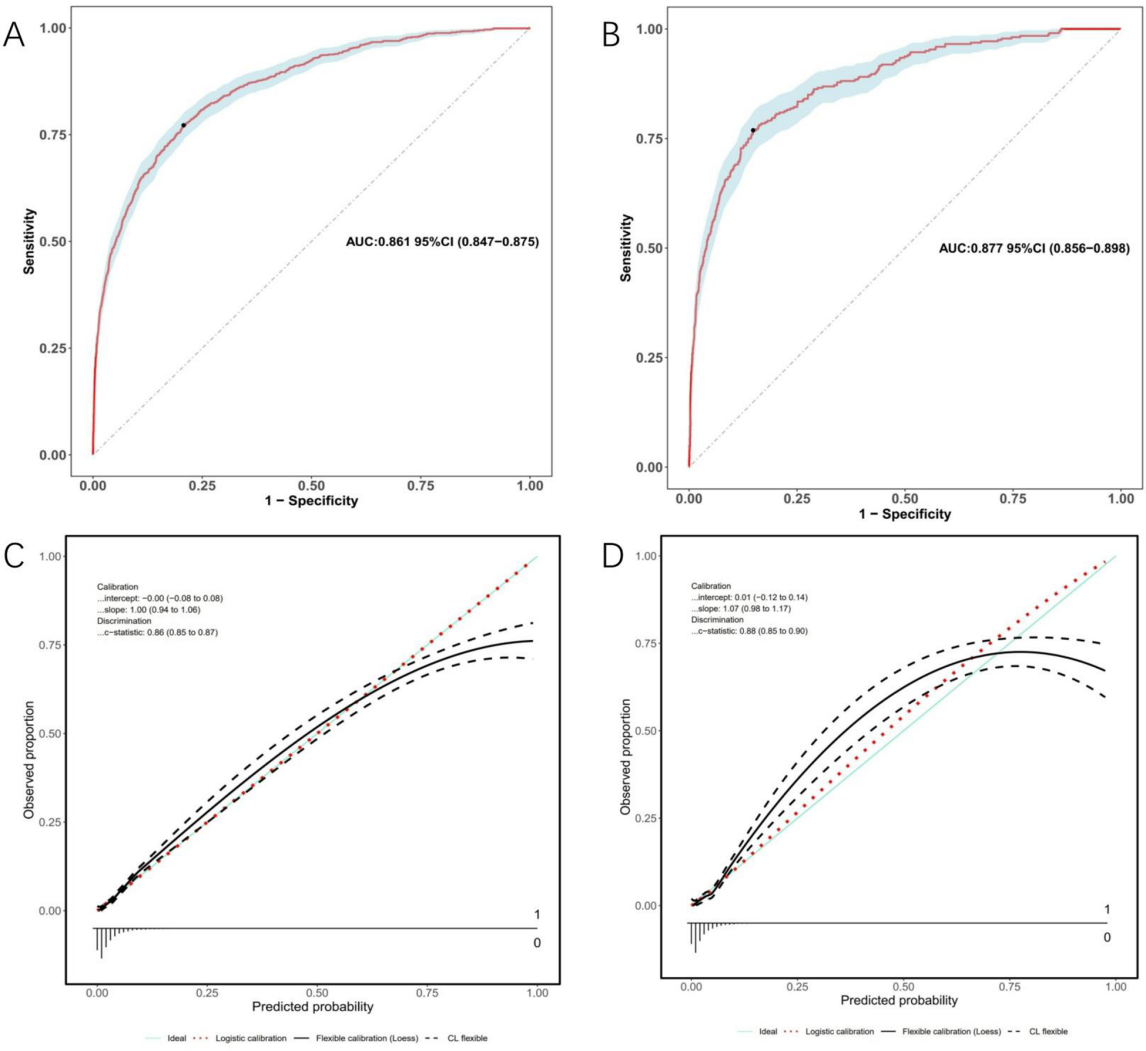
ROC curves (upper) and calibration curves (lower) of the nomogram. **(A)** ROC curves in the development cohort; **(B)** in the validation cohort. **(C)** Calibration curves in the development cohort; **(D)** in the validation cohort. Calibration curves: The *y*-axis represents the actual diagnosed cases of bleeding, while the *x*-axis represents the predicted risk of bleeding. Diagonal dotted lines represent perfect predictions by an ideal model (blue line), and the red dashed line represents the performance of the development cohort (left) and validation cohort (right). A closer alignment between the red dashed line and diagonal dotted lines indicates better prediction performance.

**Figure 7:**
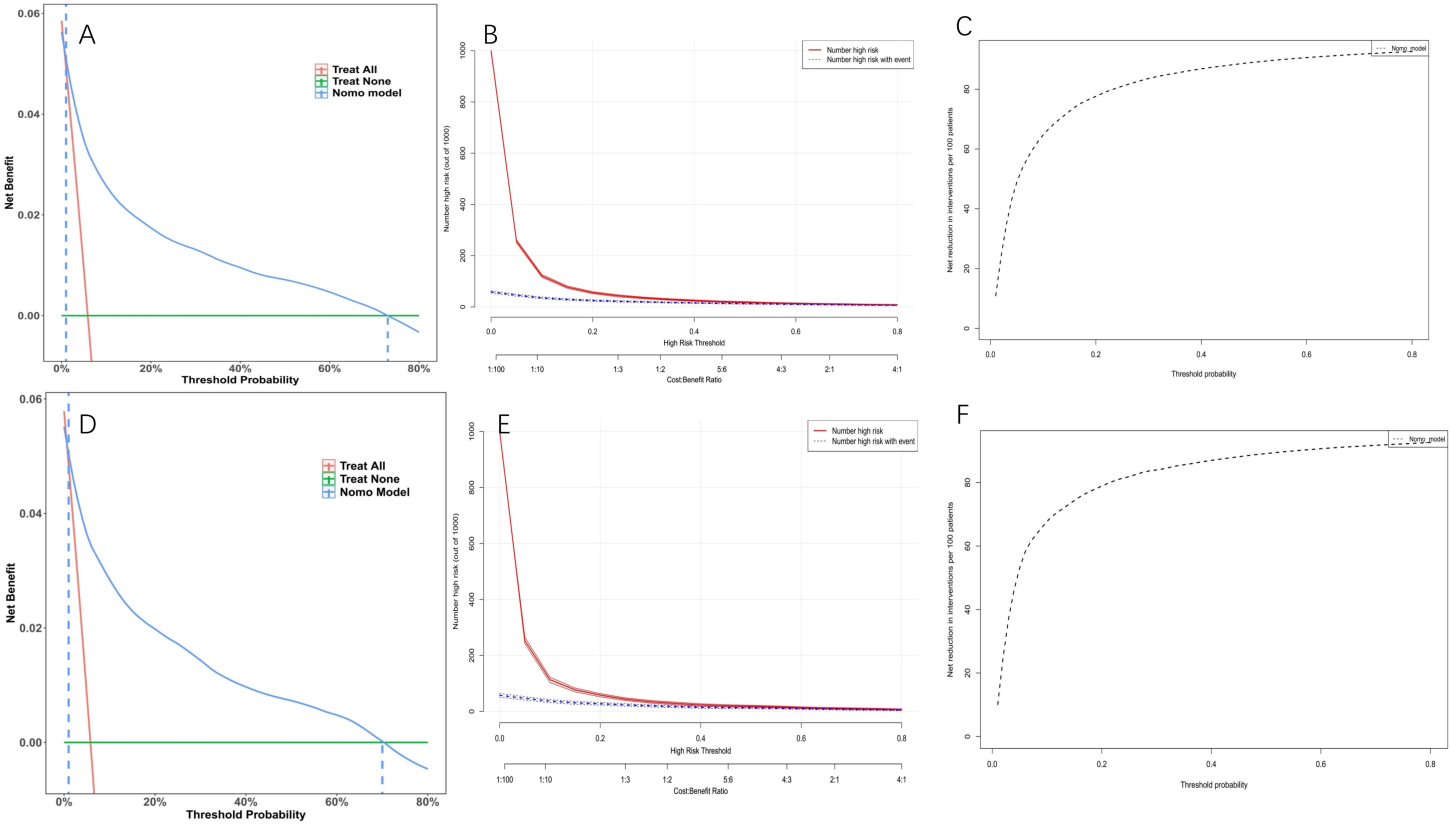
Decision curve analysis (DCA), clinical impact curves (CIC) and the net reduction curves (NRC) of the nomogram. **(A)** DCA in the development cohort; **(B)** CIC in the development cohort; **(C)** NRC in the development cohort; **(D)** DCA in the validation cohort; **(E)** CIC in the validation cohort; **(F)** NRC in the validation cohort. In the DCAs, the *y*-axis represents the net benefit. The horizontal lines labeled “None” represent the assumption that no participant experienced bleeding. The lines labeled “All” represent the assumption that all participants had bleeding. The lines labeled “nomogram model” represent the predictive model developed in this study. In CICs, the red curve represents the number of individuals classified as positive (high risk) by the model at each threshold probability, indicating the number of high-risk individuals. The blue curve represents the number of true positives (individuals with the outcome) at each threshold probability. In NRCs, the values on the *y*-axis represent the number of patients that could be reduced under the same effect size by utilizing a specific threshold probability of diagnosis, indicated by the value on the *x*-axis.

Model discrimination was assessed by calculating the AUC of the ROC curve. In Figure 6A, the AUC of the development cohort was 0.861 (95% CI 0.847–0.875), while in Figure 6B, the AUC of the validation cohort was 0.877 (95% CI 0.856–0.898). Calibration curves Figures 6C,D illustrate the excellent concordance between the predicted probability of bleeding and the actual observations in the development and validation cohort. The Hosmer and Lemeshow goodness of fit (GOF) test also showed good consistency (*p* = 0.1285). Figure 7 displays the DCA, CIC, and NRC results for the model developed in this study. The DCA results indicate that the model has a favorable net benefit in predicting bleeding risk among DAPT patients. The threshold probability ranges were 1.0%–71% for the development cohort (Figure 7A) and 1.5%–72% for the validation cohort (Figure 7D). A lower risk threshold corresponds to a higher net benefit. However, the CIC analysis revealed that as the risk threshold decreases, there is an increase in the false positive rate and unnecessary interventions (Figures 7,E). Therefore, when making final decisions, it is crucial to consider both DCA and CIC results to strike the optimal balance between high net benefit and low false positive rate. Figures 7C,F provide NRC plots, demonstrating a good fit for the development and validation cohorts of the model.

### 3.5 Model compare with single indicator

**Figure 8:**
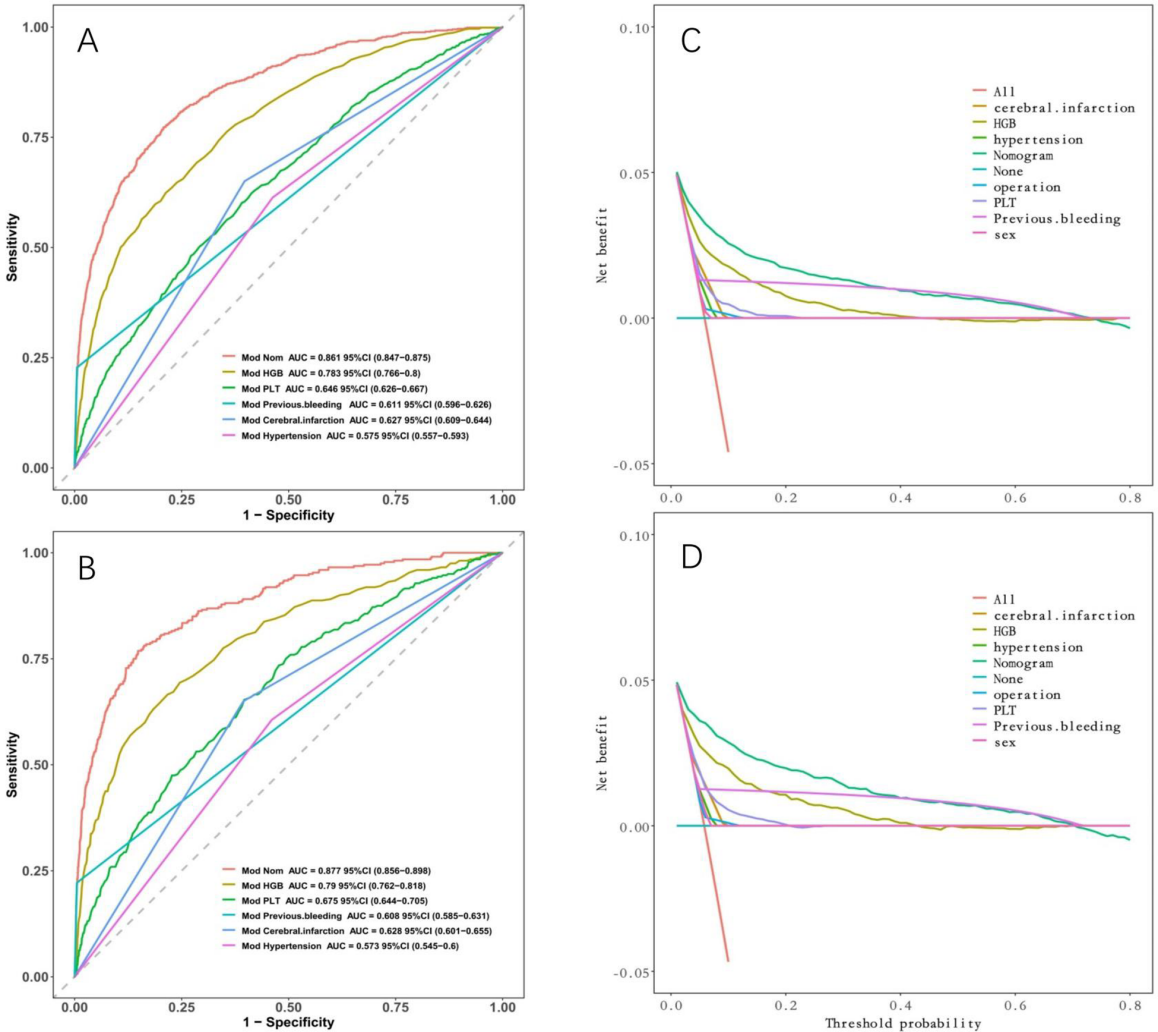
Comparison between nomogram and individual indicators. **(A)** ROC curves in the development cohort; **(B)** in the validation cohort. **(C)** DCA in the development cohort; **(D)** DCA in the validation cohort. Nomogram has the maximum AUC and maximum clinical net benefit.

We compared the discriminative ability and clinical decision-making ability of our constructed model (nomogram) with that of a single indicator. Figure 8 demonstrates that our model surpasses a single indicator in terms of both discriminative ability and clinical decision-making ability.

## 4 Discussion

In this study, we created and validated a machine learning derived model to assess bleeding risk in DAPT patients. Among the six machine learning models and LASSO regression, the XGBoost model demonstrated the highest discriminatory ability. After analyzing feature importance, we constructed the final model with seven indicators: HGB, PLT, previous bleeding, cerebral infarction, sex, surgical history, and hypertension. The derived nomogram from this model provides a visual representation that enables clinicians to estimate the bleeding risk for individual patients receiving DAPT.

Several factors have been reported to influence bleeding in DAPT patients, including HGB (20), PLT (21), previous bleeding (20), cerebral infarction (22), sex (23), Surgical history (24), and hypertension (25). Therefore, we aimed to include comprehensive information in our risk models for predicting bleeding. Previous studies have indicated that a decrease in HGB levels is a strong predictor of major bleeding (26), Low baseline HGB levels were linked to higher bleeding rates (27). Our study reaffirmed the importance of HGB as a critical indicator for assessing bleeding risk. We also identified PLT levels as another significant predictor, with lower levels indicating higher bleeding risk and poorer prognosis (28). Our predictive model aligned with previous studies’ findings. In addition, we recognize the importance of genetic components influencing platelet responses, such as the F2rl3 SNP and alterations in clopidogrel metabolism (29, 30). These genetic factors can significantly impact platelet function and response to antiplatelet therapy, potentially affecting patient outcomes. While our current study did not specifically analyze these genetic influences due to its focus on clinical and demographic data. Previous bleeding has been established as a crucial factor in guiding treatment plans for DAPT patients (20). Our risk model showed that previous bleeding increased bleeding risk in DAPT patients. Artery occlusion cerebral infarction was associated with an elevated risk of hemorrhage transformation (31), consistent with our results and potentially linked to increased antithrombotic medication usage. Moreover, male sex was independently associated with bleeding risk, in line with previous research (32). In addition to the factors mentioned above, Surgical history has been recognized as a potential predictor of bleeding (33), Our findings indicated that Surgical history is a significant factor that elevates the risk of bleeding. Consistent with prior research findings (34), our study uncovered a substantial increase in bleeding risk associated with hypertension. This elevated risk may be attributed to structural changes in blood vessels caused by hypertension, rendering them more susceptible to rupture and ultimately increasing the likelihood of bleeding (35).

Tailored management strategies are essential for DAPT patients with varying bleeding risks. High-risk patients require comprehensive evaluation and optimization. Developing an accurate predictive model for bleeding in DAPT patients holds significant clinical implications. This includes managing comorbidities, optimizing blood parameters (e.g., HGB, PLT), considering clinical features (e.g., age, sex), addressing underlying diseases (e.g., hypertension, diabetes), and evaluating medication factors to enhance coagulation function. Our developed predictive model incorporates key variables such as HGB, PLT, previous bleeding, cerebral infarction, sex, Surgical history, and hypertension using machine learning techniques like LASSO regression and XGBoost. The model's output is visualized through a user-friendly nomogram, enabling clinicians to estimate individual bleeding risk and support personalized treatment decisions. Evaluation metrics, including clinical decision curve, clinical impact curve, and net reduction curve analyses, demonstrate a high net clinical benefit, suggesting the potential for improved patient outcomes and reduced healthcare costs. We acknowledge that its performance may vary in more diverse populations. Factors such as genetic differences, variations in healthcare access, and demographic disparities can influence the effectiveness of predictive models.

Despite our significant findings, there are limitations to consider. Firstly, our study was retrospective, limiting the establishment of causal relationships. Due to the retrospective nature of this real-world study, we did not analyze data regarding BMI, estimated glomerular filtration rate, statin use, contraceptive drug use, or the influence of inflammatory markers such as CRP on our prediction model. The absence of these data limits our ability to assess their potential impacts on patient outcomes and bleeding risk. Prospective studies are needed to further validate the predictive models developed in this research. Secondly, the missing data for certain variables may have introduced bias, although we mitigated this by excluding variables with missing information in over 20% of patients. Additionally, our study focused on DAPT patients, and the generalizability of our findings to other patient populations requires further investigation.

Overall, our model offers valuable clinical insights and aids in decision-making for managing bleeding in DAPT patients. It has the potential to optimize treatment planning, improve patient outcomes, and enhance resource utilization. In future research, external validation of our models in different patient cohorts is warranted to confirm their reliability and effectiveness in routine clinical practice. Moreover, the integration of additional variables and the exploration of different machine learning algorithms could further improve the accuracy of bleeding risk prediction. Finally, prospective studies evaluating the impact of utilizing our predictive models in clinical decision-making would provide valuable insights into their potential benefits and limitations.

## Data Availability

The raw data supporting the conclusions of this article will be made available by the authors, without undue reservation.
