# Supplementary figures and images for: Machine learning derived model for the prediction of bleeding in dual antiplatelet therapy patients
Source: Front Cardiovasc Med. 2024 Oct 2;11:1402672. doi: 10.3389/fcvm.2024.1402672 (PMC11479971; doi:10.3389/fcvm.2024.1402672)

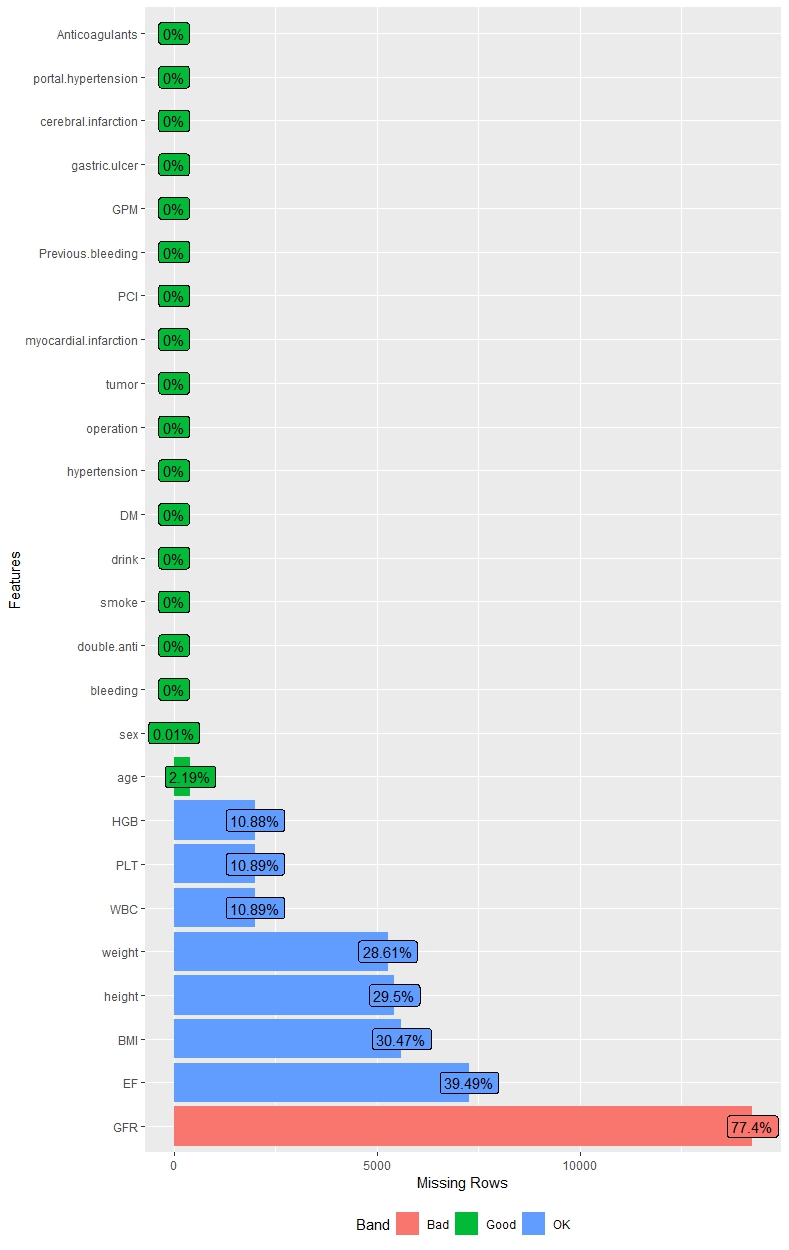

Supplement: Supplementary file 1 [file Image1.jpeg]
